# Effectiveness of Acupressure on Anxiety, Fatigue, and Sleep Quality Among Operating Room Personnel: A Randomized Controlled Trial

**DOI:** 10.1002/hsr2.72680

**Published:** 2026-06-19

**Authors:** Rana Abjar, Ebrahim NasiriFormi, Amir Hooman Kazemi, Hooshang Akbari, Leila Sadati

**Affiliations:** ^1^ Department of Anesthesiology and Operating Room, Student research Committee, School of Allied Sciences Mazandaran University of Medical Sciences Sari Iran; ^2^ Department of Operating Room, School of Paramedical Sciences Alborz University of Medical Sciences Karaj Iran; ^3^ Department of Anesthesiology, Operating room, School of Allied Medical Sciences, Traditional and Complementary Medicine Research Center Addiction Institute Sari Mazandaran Iran; ^4^ Department of Traditional Medicine, School of Persian Medicine Tehran University of Medical Sciences Tehran Iran; ^5^ International School Beijing University of Chinese Medicine Beijing China; ^6^ Department of Anesthesiology, Operating room, School of Allied Medical Sciences Mazandaran University of Medical Sciences Sari Iran

**Keywords:** acupressure, anxiety, complementary therapies, fatigue, operating rooms, sleep

## Abstract

**Background and Aims:**

High‐quality sleep, calmness, and lack of fatigue are vital for the health of critical hospital staff. Operating room personnel experience significant mental and physical stress, including anxiety and sleep disturbances. This study evaluates acupressure, a traditional complementary therapy, for reducing anxiety and exhaustion and improving sleep quality among OR staff at baseline.

**Methods:**

A clinical experiment with random allocation was conducted involving 70 operating room personnel experiencing at least two of the following conditions: anxiety, exhaustion, or sleep quality. Participants were allocated randomly to either an intervention group receiving acupressure or a comparison group undergoing simulated acupressure over a period of 4 weeks. The control group received simulated acupressure involving light touching near the acupoints without pressure. Standardized tools were utilized to measure anxiety, exhaustion, and sleep condition at the initial stage and at intervals of two and 4 weeks following the intervention. Data were analyzed using SPSS 22.

**Results:**

At baseline, no notable disparities were evident among the groups regarding anxiety or fatigue levels. Nevertheless, following the intervention, meaningful differences became apparent, favoring the acupressure cohort (*p *< 0.001). Likewise, a considerable variation in sleep condition trends was identified over time (*p *< 0.001), with the acupressure cohort demonstrating a marked enhancement in sleep condition.

**Conclusion:**

Acupressure demonstrates a significant positive effect in reducing anxiety and fatigue and improving sleep quality among operating room staff. These results endorse acupressure as an alternative, non‐pharmacological strategy for addressing stress‐related conditions in high‐pressure work environments.

## 1 Introduction

High‐quality sleep, calmness, and the absence of fatigue are essential pillars of human health, especially for personnel in critical hospital departments. The operating room is one of the most complex and demanding work environments in the healthcare system, where patients undergo critical stages of their treatment. Consequently, ensuring patient health and safety is paramount. A crucial factor in achieving this is the health and mental well‐being of operating room staff. High workload, the nature of the work, risk of needlestick injuries and cuts, a challenging physical environment, variable working hours, the need for immediate decision‐making, disproportionate income relative to job demands, and insufficient time off are among the significant stressors faced by operating room personnel [1]. Stress and anxiety over time cause fatigue [2] and decreased sleep quality [3, 4], which can lead to reduced attention and increased errors, resulting in failure to adhere to standard principles of safe patient care [5]. Also, sleep disorders and related problems affect the performance of duties in the workplace and increase the negative consequences such as occupational accidents, absenteeism, etc [6]. In one study, nurses with high sleep disturbances had a 3.26‐fold chance of occurrence of nursing errors [7]. Therefore, considering the potential influence of anxiety, exhaustion, and inadequate sleep quality on the occurrence and intensity of medical errors, there is a vital need for meticulous program design and the implementation of effective measures to address these challenges among operating room personnel, thereby minimizing adverse outcomes. Both medicinal and alternative interventions are available to manage these disorders [8]. Although the use of medications to treat anxiety and sleep disorders is common, in most cases, these drugs are associated with several side effects, including dependence. As a result, Complementary and non drug therapies are gaining widespread acceptance among healthcare professionals due to their holistic approach and minimal side effects [9]. One of these non pharmacological interventions is the use of acupressure. Acupressure is a type of massage performed on 365 special energetic points along 12 major pathways, aiming to promote health and provide treatment. Acupressure is a complementary and alternative medicine (CAM) technique rooted in Traditional Chinese Medicine (TCM) and Persian Traditional Medicine (PTM). It involves applying gentle but firm pressure with the fingertips to specific points on the skin called acupoints, which lie along energy pathways known as meridians. According to TCM, vital energy or Qi flows through these meridians, and its balance is essential for maintaining health and well‐being. Acupressure aims to restore this balance, thereby promoting physical and mental health. This non‐invasive therapy is safe, portable, and easy to learn. Numerous studies have demonstrated its effectiveness in reducing stress, anxiety, fatigue, and improving sleep quality. Although the exact biological mechanisms are not fully understood, it is believed that acupressure modulates the autonomic nervous system, influencing physiological responses such as heart rate and skin conductance. Despite positive findings, further research is necessary to establish optimal treatment protocols and to explore its benefits among specific high‐stress populations, such as healthcare workers. This study aims to examine the impact of acupressure on anxiety, exhaustion, and sleep disturbances among operating room personnel [10]. Numerous investigations have demonstrated the impact of acupressure in alleviating patient exhaustion, enhancing sleep conditions, and mitigating anxiety [11, 12]. Although numerous investigations have demonstrated the impact of acupressure on alleviating patient exhaustion, enhancing sleep conditions, and mitigating anxiety, there remains a notable scarcity of rigorous trials focusing specifically on healthcare workers, particularly those in high‐pressure environments such as operating rooms. Healthcare personnel experience unique occupational stressors and health challenges distinct from those of patients, underscoring the need for dedicated research in this population. Given the scarcity of research exploring the application of acupressure among healthcare workers, particularly those in high‐pressure settings such as operating rooms, the purpose of this study is to evaluate the impact of acupressure on anxiety, exhaustion, and sleep disturbances among operating room personnel.

## 2 Materials and Methods

### 2.1 Research Framework and Participants

The current research represents a randomized clinical experiment conducted after securing ethical clearance and adhering to the standards set forth by the Declaration of Helsinki and national ethical protocols. The trial was officially documented in the Clinical Trial Registry under the registration code (IRCT20200305046701N1). A total of 70 staff members employed in the operating rooms of hospitals in Karaj were enrolled as study participants.

### 2.2 Inclusion Criteria Included

A group of surgical and anesthesia staff working in operating rooms was chosen according to the study's defined eligibility and disqualification standards Individuals eligible for the study needed to satisfy at least two criteria among the following: experiencing fatigue ranging from moderate to severe, anxiety levels from mild to severe, or having poor sleep quality. Additional inclusion criteria included the absence of wounds or abrasions in the target areas of the acupressure points, no history of acupuncture or acupressure within the past month, no use of sedatives or neuroleptics within the past month, no use of anticoagulant or antiplatelet drugs, no systemic diseases, not being pregnant, and willingness to participate in the study. Qualified individuals were distributed into the intervention and control groups through a process of random allocation.

### 2.3 Randomization and Blinding

A random allocation sequence for 70 employees, who met the inclusion and exclusion criteria, was created using computer‐generated random numbers to allocate them to two intervention and control groups. Allocation concealment was achieved through sealed, sequentially numbered opaque envelopes that were only opened at the start of the intervention and allocation to avoid selection bias. Because all participants were from a city and center, blinding them was difficult. The observer collecting the data relied solely on the standardized data collection forms and questionnaires and did not perform any interventions during data collection. No adverse events or side effects were reported during the trial period. All participants tolerated the intervention well without any complications.

### 2.4 Data Collection Tool

This study employed three validated questionnaires to collect data on fatigue, anxiety, and sleep quality: the Multidimensional Fatigue Inventory (MFI), the Pittsburgh Sleep Quality Index (PSQI), and the Beck Anxiety Inventory (BAI).

The BAI consists of 21 items, each rated on a 4‐point Likert scale from 0 (not at all) to 3 (severely). The total score ranges from 0 to 63 and is used to categorize anxiety severity as follows: 0–7 indicates minimal or no anxiety, 8–15 mild anxiety, 16–25 moderate anxiety, and 26–63 severe anxiety.

The PSQI is a self‐administered Standard questionnaire comprising 18 items that evaluate sleep quality over the past month across seven components: subjective sleep quality, sleep latency, sleep duration, habitual sleep efficiency, sleep disturbances, use of sleep medication, and daytime dysfunction. Each component is scored from 0 to 3, with a total global score ranging from 0 to 21. A total score greater than 5 indicates poor sleep quality.

The MFI is a 20‐item questionnaire assessing five dimensions of fatigue: general fatigue, physical fatigue, mental fatigue, reduced activity, and reduced motivation. Each dimension consists of four items rated on a 5‐point Likert scale, with total scores ranging from 20 to 100. Fatigue severity in this study was categorized based on total scores as follows: 20–46 mild fatigue, 47–73 moderate fatigue, and 74–100 severe fatigue.

Using these standardized and validated tools, we objectively classified participants' fatigue, anxiety, and sleep quality levels according to established cutoff scores, ensuring reliable and reproducible measurement.

### 2.5 Sampling

The sample size for each group was determined using G‐Power software and a one‐way ANOVA for two groups, with an alpha of 0.05, 90% power, and a large effect size. This calculation indicated that 28 participants per group were needed. To compensate for an expected 20% dropout, 35 participants were enrolled in each group [13].

### 2.6 Intervention

After the initial assessment of anxiety, fatigue, and sleep disorders in both groups, the intervention group received instructions on acupressure techniques through educational videos, which were also provided as reminders. Participants were asked to apply circular pressure on designated acupuncture points (DU20, Gallbladder20, Liver3, Kidney3, DU24, Ren17, and Stomach36) for 1–2 min per point, three times daily, with pressure applied up to participant tolerance (maximum 3 kilograms), over a period of 4 weeks [12, 13, 14]. The control group was trained to touch points near the acupressure locations without applying pressure(simulated acupressure), meaning no acupressure was performed. They also did not receive the educational videos. Daily follow‐ups via telephone and virtual communication were conducted to ensure adherence. Reassessments of anxiety, fatigue, and sleep disorders were conducted at two and 4 weeks after the intervention.

### 2.7 Data Analysis

The dataset was processed with SPSS software, version 22. To summarize the data, means and standard deviations were computed. The Kolmogorov‐Smirnov test was applied to evaluate the normality of the variables. Based on the distribution results, either independent samples t‐tests or Mann‐Whitney *U* tests were conducted to compare numerical variables. For demographic variables, categorical data such as gender and marital status were analyzed using Chi‐square tests, whereas continuous variables like age and work experience were evaluated with independent samples t‐tests. Fatigue and sleep quality variables, measured repeatedly over time, were analyzed using repeated measures Analysis of Variance (ANOVA) to assess group differences and changes across multiple time points. Anxiety levels were examined quantitatively using repeated measures ANOVA, and qualitatively with Chi‐square tests. Sleep, fatigue, and anxiety scores were also compared using an independent t‐test. The analysis followed an intention‐to‐treat (ITT) principle, with statistical significance determined at a threshold of *p *< 0.05. The statistical tests were conducted as one‐sided tests. Data were analyzed using IBM SPSS Statistics software, version 22.

## 3 Results

### 3.1 Participants' Demographics

**Figure 1:**
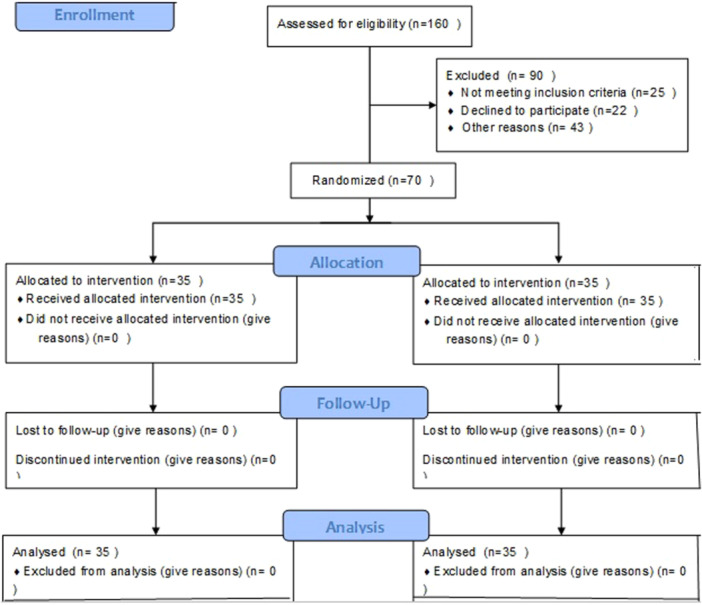
CONSORT flow diagram.

The results of our study showed that 70 participants were allocated into two intervention groups, acupressure and control, and completed the study over a period of 4 weeks, with no exclusions (Figure 1). In this study, 70 operating room staff participated, including 59/70 males (84.3%) and 11/70 females (15.7%). Regarding gender,85.7% of the acupressure group were male and 14.3% (female, while in the control group, 82.9% were male and 17.1% female, with no significant difference between the groups (Chi‐square test, *p *> 0.05). The mean age in the control group was 30.77 ± 8.14 years, while in the intervention group it was 27.11 ± 7.54 years. The difference in mean age between the two groups was not statistically significant (Independent samples t‐test, *p *< 0.06). The mean work experience in the control group was 8.05 years with a standard deviation of 6.54 and in the intervention group was 6.88 years with a standard deviation of 3.79. The Independent samples t‐test showed that the difference in mean work experience between the two groups was not statistically significant (*p *< 0.4). Of the 35 people in the intervention group, 24/35 (68.6%) were single, compared to 22/35 (62.9%) in the control group.

### 3.2 Fatigue

Repeated measures Analysis of Variance (ANOVA) showed no significant difference in fatigue levels between the two groups prior to the intervention (*p *< 0.675). However, significant changes appeared at 2 weeks (*p *< 0.001) and 4 weeks (*p *< 0.001) after the intervention, with mean fatigue scores significantly declining over time in the intervention group, whereas the control group showed no reduction (Table 1).

**Table 1:** Comparison of the mean scores of total fatigues between two groups.

Time	Acupressure (*n* = 35)				Control (*n* = 35)				*p* value by t‐test	CI 95%
	Mean	SD	Max	Min	Mean	SD	Max	Min		Mean difference
Before	59.12	7.77	85	49	62.90	8.37	86	48	0.067	−3.78 (−7.64, 0.08)
2 weeks later	53.58	7.9	80	45	63	8.16	86	49	< 0.001	−9.42 (−13.26, −5.58)
4 weeks later	50.09	6.32	70	43	64.03	7.40	84	49	< 0.001	−13.94 (−17.22, −10.66
*p*‐value by repeated measures	< 0.001									

Among the dimensions of fatigue, only the rates of general and physical fatigue in the acupressure group were significantly reduced at two and 4 weeks after the intervention (*p *< 0.001). No significant decrease was observed in the control group. At both two and 4 weeks after the intervention, a significant difference was found between the groups regarding reduced activity and motivation. The average scores for motivational fatigue declined in the intervention group but rose in the control group. Mental fatigue showed no significant difference between groups at baseline (*p *< 0.72) and at 2 weeks after the intervention (*p *< 0.28); however, a significant reduction in this dimension was noted in the intervention group at 4 weeks after the intervention (*p *< 0.001).

### 3.3 Anxiety

Repeated measures ANOVA revealed that the mean anxiety score significantly decreased in the intervention group, whereas the control group showed no change (Table 2). Qualitative anxiety analysis revealed no significant difference at baseline (*p *> 0.92), but significant differences emerged at 2 weeks (*p *> 0.03) and 4 weeks (*p *< 0.001), with the number of participants reporting no anxiety increasing to 18 (62%) in the acupressure group after 4 weeks.

**Table 2:** Comparison of mean anxiety scores across between two groups period.

Time	Anxiety				Anxiety				*p* value by t‐test	CI 95%
	Acupressure, (*n* = 35)				Control (*n* = 35)					
	Mean	SD	Max	Min	Mean	SD	Max	Min		Mean difference
Before	14.86	6.95	33	8	15.25	4.97	27	8	0.81	−0.39 (−3.29, 2.51)
2 weeks later	12.51	5.93	26	6	15.14	4.18	24	9	0.059	−2.63 (−5.07, −0.19)
4 weeks later	10.10	5.17	24	4	15.78	4.49	24	9	< 0.001	−5.68 (−8.00, −3.36)
*p*‐value by repeated measures	< 0.001									

### 3.4 Sleep Quality

Repeated measures ANOVA showed that the trend of the mean total sleep quality score over time was significantly different between the groups (Table 3), with a significant improvement in the intervention group and a worsening in the control group. At baseline, there was no significant difference between the two groups regarding subjective sleep quality. However, subjective sleep quality was notably improved at two and 4 weeks. Comparison of actual sleep scores (0–3) revealed no significant differences between the groups before or after the intervention; however, a significant effect of time was observed. Comparison of sleep latency scores (0–5) showed no significant difference between the groups at baseline, but a significant difference was observed after the intervention. Scores for sleep disturbances and daytime dysfunction (0–3) did not differ significantly between groups at baseline or after 2 weeks, but a significant difference emerged at 4 weeks. When comparing sleep efficiency percentages, no significant differences were found between groups before or after the intervention; however, the effect of time differed significantly between groups (*p* > 0.001). Also, no participants reported using sleeping pills at baseline, 2 weeks, or at 4 weeks post‐intervention.

**Table 3:** Comparison of mean sleep quality scores between two groups.

Time	Acupressure (*n* = 35)				Control (*n* = 35)				*p* value by t‐test	CI 95%
	Mean	SD	Max	Min	Mean	SD	Max	Min		Mean differences
Before	8.22	2.30	14	6	9.65	2.53	16	6	0.03	−1.43 (−2.59, −0.27)
2 weeks later	8.59	1.98	13	6	11.06	2.38	15	6	< 0.001	−2.47 (−3.51, −1.43)
4 weeks later	7.44	1.52	11	5	11.06	2.32	15	6	< 0.001	−3.62 (−4.56, −2.68)
*p*‐value by repeated measures	< 0.001									

## 4 Discussion

The study included 70 operating room staff divided into acupressure and control groups with comparable demographic characteristics. No significant differences were observed between the groups in fatigue, anxiety, or sleep quality at baseline. Following the intervention, the acupressure group demonstrated significant improvements, with reduced general and physical fatigue, decreased anxiety levels, and enhanced sleep quality over 4 weeks. In contrast, the control group showed no improvement or even worsening trends in these outcomes. These findings suggest that acupressure positively affects fatigue, anxiety, and sleep quality among operating room staff. In the present study, the level of anxiety among operating room staff in the acupressure group was significantly reduced compared to the control group, with mean anxiety scores decreasing from 14.86 at baseline to 10.10 at 4 weeks post‐intervention. These results align with the findings of Ajorpaz et al [14]. In the study by Mousavi et al., acupressure decreased the mean of overt and covert anxiety in midwifery students, while anxiety increased in nursing and operating room students [15], which may be related to the characteristics of the samples and the conditions for applying these interventions. Despite differences in participants, the results of the present study align with the findings of Shahdadi et al [16]. They investigated the effect of acupressure on anxiety and quality of life in patients with type 2 diabetes. Their study demonstrated a significant reduction in total anxiety scores, decreasing from a moderate level before the intervention to a mild level afterward, alongside improvements in all anxiety subscales and overall quality of life. Shahdadi et al. reported that the mean total anxiety score significantly decreased from 111.21 ± 17.82 before the intervention to 78.91 ± 9.13 after acupressure treatment. These results support the positive impact of acupressure on reducing anxiety and enhancing quality of life, consistent with our study findings. Agarwal et al. demonstrated that acupressure applied significantly reduced pre‐operative anxiety and bispectral index (BIS) values during the intervention period compared to the control group. Anxiety levels decreased notably from a median of 8 at baseline to 5 after 10 min of acupressure but returned to baseline within 30 min after pressure release. Similarly, BIS values were significantly lower during acupressure application, indicating reduced stress and arousal. These findings highlight the short‐term effectiveness of acupressure as a non‐pharmacological method to alleviate preoperative anxiety [16, 17]. Other studies have also demonstrated the positive effects of acupressure on reducing anxiety in patients [18, 19]. In the present study, the level of anxiety among operating room staff in the acupressure group was significantly reduced compared to the control group, with mean anxiety scores decreasing from 14.86 at baseline to 10.10 at 4 weeks post‐intervention. These results align with the findings of Ajorpaz et al. In the study by Mousavi et al., acupressure decreased the mean of overt and covert anxiety in midwifery students, while anxiety increased in nursing and operating room students. This difference may be related to variations in sample characteristics such as participants’ age, professional background, and baseline anxiety levels, as well as differences in the conditions for applying the interventions, including duration, frequency, and settings of acupressure sessions. Despite these differences, the results of the present study align with previous findings, supporting the beneficial effects of acupressure in reducing anxiety among healthcare staff.‬‬‬‬‬‬‬‬‬‬‬‬‬‬‬‬‬‬‬‬‬‬‬‬‬‬‬‬‬‬‬‬‬‬‬‬‬‬‬‬‬‬‬‬‬‬‬‬‬‬‬‬‬‬‬‬‬‬‬‬‬‬‬‬‬‬‬‬‬‬‬‬‬‬‬‬‬‬‬‬‬‬‬‬‬‬‬‬‬‬‬‬‬‬‬‬‬‬‬‬‬‬‬‬‬‬‬‬‬‬‬‬‬‬‬‬‬‬‬‬‬‬‬‬‬‬‬‬‬‬‬‬‬‬‬‬‬‬‬‬‬‬‬‬‬‬‬‬‬‬‬‬‬‬‬‬‬‬‬‬‬‬‬‬‬‬‬‬‬‬‬‬‬‬‬‬‬‬‬‬‬‬‬‬‬‬‬‬‬‬‬‬‬‬‬‬‬‬‬‬‬‬‬‬‬‬‬‬‬‬‬‬‬‬‬‬‬‬‬‬‬‬‬‬‬‬‬‬‬‬‬‬‬‬‬‬‬‬‬‬‬‬‬‬‬‬‬‬‬‬‬‬‬‬‬‬‬‬‬‬‬‬‬‬‬‬‬‬‬‬‬‬‬‬‬‬‬‬‬‬‬‬‬‬‬‬‬‬‬‬‬‬‬‬‬‬‬‬‬‬‬‬‬‬‬‬‬‬‬‬‬‬‬‬‬‬‬‬‬‬‬‬‬‬‬‬‬‬‬‬‬‬‬‬‬‬‬‬‬‬‬‬‬‬‬‬‬‬‬‬‬‬‬‬‬‬‬‬‬‬‬‬‬‬‬‬‬‬‬‬‬‬‬‬‬‬‬‬‬‬‬‬‬‬‬‬‬‬‬‬‬‬‬‬‬‬‬‬‬‬‬‬‬‬‬‬‬‬‬‬‬‬‬‬‬‬‬‬‬‬‬‬‬‬‬‬‬‬‬‬‬‬‬‬‬‬‬‬‬‬‬‬‬‬‬‬‬‬‬‬‬‬‬‬‬‬‬‬‬‬‬‬‬‬‬‬‬‬‬‬‬‬‬‬‬‬‬‬‬‬‬‬‬‬‬‬‬‬‬‬‬‬‬‬‬‬‬‬‬‬‬‬‬‬‬‬‬‬‬‬‬‬‬‬‬‬‬‬‬‬‬‬‬‬‬‬‬‬‬‬‬‬‬‬‬‬‬‬‬‬‬‬‬‬‬‬‬‬‬‬‬‬‬‬‬‬‬‬‬‬‬‬‬‬‬‬‬‬‬‬‬‬‬‬‬‬‬‬‬‬‬‬‬‬‬‬‬‬‬‬‬‬‬‬‬‬‬‬‬‬‬‬‬‬‬‬‬‬‬‬‬‬‬‬‬‬‬‬‬‬‬‬‬‬‬‬‬‬‬‬‬‬‬‬‬‬‬‬‬‬‬‬‬‬‬‬‬‬‬‬‬‬‬‬‬‬‬‬‬‬‬‬‬‬‬‬‬‬‬‬‬‬‬‬‬‬‬‬‬‬‬‬‬‬‬‬‬‬‬‬‬‬‬‬‬‬‬‬‬‬‬‬‬‬‬‬‬‬‬‬‬‬‬‬‬‬‬‬‬‬‬‬‬‬‬‬‬‬‬‬‬‬‬‬‬‬‬‬‬‬‬‬‬‬‬‬‬‬‬‬‬‬‬‬‬‬‬‬‬‬‬‬‬‬‬‬‬‬‬‬‬‬‬‬‬‬‬‬‬‬‬‬‬‬‬‬‬‬‬‬‬‬‬‬‬‬‬‬‬‬‬‬‬‬‬‬‬‬‬‬‬‬‬‬‬‬‬‬‬‬‬‬‬‬‬‬‬‬‬‬‬‬‬‬‬‬‬‬‬‬‬‬‬‬‬‬‬‬‬‬‬‬‬‬‬‬‬‬‬‬‬‬‬‬‬‬‬‬‬‬‬‬‬‬‬‬‬‬‬‬‬‬‬‬‬‬‬‬‬‬‬‬‬‬‬‬‬‬‬‬‬‬‬‬‬‬‬‬‬‬‬‬‬‬‬‬‬‬‬‬‬‬‬‬‬‬‬‬‬‬‬‬‬‬‬‬‬‬‬‬‬‬‬‬‬‬‬‬‬‬‬‬‬‬‬‬‬‬‬‬‬‬‬‬‬‬‬‬‬‬‬‬‬‬‬‬‬‬‬‬‬‬‬‬‬‬‬‬‬‬‬‬‬‬‬‬‬‬‬‬‬‬‬‬‬‬‬‬‬‬‬‬‬‬‬‬‬‬‬‬‬‬‬‬‬‬‬‬‬‬‬‬‬‬‬‬‬‬‬‬‬‬‬‬‬‬‬‬‬‬‬‬‬‬‬‬‬‬‬‬‬‬‬‬‬‬‬‬‬‬‬‬‬‬‬‬‬‬‬‬‬‬‬‬‬‬‬‬‬‬‬‬‬‬‬‬‬‬‬‬‬‬‬‬‬‬‬‬‬‬‬‬‬‬‬‬‬‬‬‬‬‬‬‬‬‬‬‬‬‬‬‬‬‬‬‬‬‬‬‬‬‬‬‬‬‬‬‬‬‬‬‬‬‬‬‬‬‬‬‬‬‬‬‬‬‬‬‬‬‬‬‬‬‬‬‬‬‬‬‬‬‬‬‬‬‬‬‬‬‬‬‬‬‬‬‬‬‬‬‬‬‬‬‬‬‬‬‬‬‬‬‬‬‬‬‬‬‬‬‬‬‬‬‬‬‬‬‬‬‬‬‬‬‬‬‬‬‬‬‬‬‬‬‬‬‬‬‬‬‬‬‬‬‬‬‬‬‬‬‬‬‬‬‬‬‬‬‬‬‬‬‬‬‬‬‬‬‬‬‬‬‬‬‬‬‬‬‬‬‬‬‬‬‬‬‬‬‬‬‬‬‬‬‬‬‬‬‬‬‬‬‬‬‬‬‬‬‬‬‬‬‬‬‬‬‬‬‬‬‬‬‬‬‬‬‬‬‬‬‬‬‬‬‬‬‬‬‬‬‬‬‬‬‬‬‬‬‬‬‬‬‬‬‬‬‬‬‬‬‬‬‬‬‬‬‬‬‬‬‬‬‬‬‬‬‬‬‬‬‬‬‬‬‬‬‬‬‬‬‬‬‬‬‬‬‬‬‬‬‬‬‬‬‬‬‬‬‬‬‬‬‬‬‬‬‬‬‬‬‬‬‬‬‬‬‬‬‬‬‬‬‬‬‬‬‬‬‬‬‬‬‬‬‬‬‬‬‬‬‬‬‬‬‬‬‬‬‬‬‬‬‬‬‬‬‬‬‬‬‬‬‬‬‬‬‬‬‬‬‬‬‬‬‬‬‬‬‬‬‬‬‬‬‬‬‬‬‬‬‬‬‬‬‬‬‬‬‬‬‬‬‬‬‬‬‬‬‬‬‬‬‬‬‬‬‬‬‬‬‬‬‬‬‬‬‬‬‬‬‬‬‬‬‬‬‬‬‬‬‬‬‬‬‬‬‬‬‬‬‬‬‬‬‬‬‬‬‬‬‬‬‬‬‬‬‬‬‬‬‬‬‬‬‬‬‬‬‬‬‬‬‬‬‬‬‬‬‬‬‬‬‬‬‬‬‬‬‬‬‬‬‬‬‬‬‬‬‬‬‬‬‬‬‬‬‬‬‬‬‬‬‬‬‬‬‬‬‬‬‬‬‬‬‬‬‬‬‬‬‬‬‬‬‬‬‬‬‬‬‬‬‬‬‬‬‬‬‬‬‬‬‬‬‬‬‬‬‬‬‬‬‬‬‬‬‬‬‬‬‬‬‬‬‬‬‬‬‬‬‬‬‬‬‬‬‬‬‬‬‬‬‬‬‬‬‬‬‬‬‬‬‬‬‬‬‬‬‬‬‬‬‬‬‬‬‬‬‬‬‬‬‬‬‬‬‬‬‬‬‬‬‬‬‬‬‬‬‬‬‬‬‬‬‬‬‬‬‬‬‬‬‬‬‬‬‬‬‬‬‬‬‬‬‬‬‬‬‬‬‬‬‬‬‬‬‬‬‬‬‬‬‬‬‬‬‬‬‬‬‬‬‬‬‬‬‬‬‬‬‬‬‬‬‬‬‬‬‬‬‬‬‬‬‬‬‬‬‬‬‬‬‬‬‬‬‬‬‬‬‬‬‬‬‬‬‬‬‬‬‬‬‬‬‬‬‬‬‬‬‬‬‬‬‬‬‬‬‬‬‬‬‬‬‬‬‬‬‬‬‬‬‬‬‬‬‬‬‬‬‬‬‬‬‬‬‬‬‬‬‬‬‬‬‬‬‬‬‬‬‬‬‬‬‬‬‬‬‬‬‬‬‬‬‬‬‬‬‬‬‬‬‬‬‬‬‬‬‬‬‬‬‬‬‬‬‬‬‬‬‬‬‬‬‬‬‬‬‬‬‬‬‬‬‬‬‬

The second variable studied was sleep quality. Our results showed a decreasing trend of mean scores over time in the intervention group. A quasi‐experimental study examining the effect of acupressure on sleep quality in 40 diabetic patients reported that the mean sleep quality score improved from 8.19 ± 3.92 before the intervention to 4.17 ± 2.15 after 4 weeks. The study also indicated a significant difference in sleep quality before and after the intervention across all measures [20]. However, in our study, despite the decrease in the mean score and improved sleep quality, scores did not reach a range indicative of good sleep, even after 4 weeks. This suggests that a longer intervention period may be necessary. An integrative review published in 2019 examining the effects of acupressure on sleep quality, depression, anxiety, and distress in older adults found that nine studies consistently reported beneficial effects of acupressure on sleep quality, which aligns with our findings [11]. In the study by Shariati et al., a significant difference was observed between the acupressure group and the control group in total PSQI scores and all sleep quality indicators after 4 weeks of intervention in hemodialysis patients. Despite differences in the samples and acupressure points, the result in the total score is consistent with our study [21]. The review found that acupressure was effective for several symptoms, particularly for nausea and vomiting, pain, dyspnea, fatigue, and insomnia, though the authors noted the need for more rigorous studies to confirm these findings

A systematic review of 30 articles, including 2363 participants, evaluating the effect of acupressure on improving sleep quality in people with insomnia showed that acupressure is superior to both placebo and pharmacotherapy in improving sleep quality [22]. Although previous systematic review used sham acupressure as the control, in our study, the control group only received light touch near the acupressure points without any pressure. Despite this difference, our results are consistent with the systematic review, supporting the positive effects of acupressure.

The third variable evaluated was fatigue. The results of our study demonstrated that the rate of fatigue in the acupressure group decreased significantly compared to the control group, with the mean severity of fatigue improving from 59.12 before the intervention to 50.9 after 4 weeks. A systematic review by Lee and Frazier (2011) examined randomized controlled trials on the effectiveness of acupressure for managing symptoms such as nausea and vomiting, pain, respiratory distress, fatigue, and insomnia across various patient groups. The results of six studies included in this review indicated a positive effect of acupressure on decreasing fatigue and reducing insomnia in different populations [10], which aligns with our findings. A study examining the effect of acupressure on the fatigue of 85 cancer patients for 10 days demonstrated that acupressure in the intervention group had a positive effect on patients' fatigue [23]. The cited study observed the effect of acupressure after a shorter period (5 and 10 days), while the present study investigated the effect of acupressure over a longer period, indicating the rapid and continuous effect on reducing the severity of fatigue. The observed reductions in fatigue and anxiety scores, along with improved sleep quality in the intervention group, exceed minimal clinically important difference thresholds reported in previous research. These findings indicate that the acupressure intervention produces meaningful clinical benefits, enhancing personnels' overall quality of life beyond mere statistical significance. In this study, no participant exclusions occurred after allocation, and all 70 participants completed the intervention and follow‐up, minimizing any risk of attrition bias. This high retention rate was ensured through continuous engagement and support, thereby enhancing the reliability and validity of the study findings. This study's strengths include a randomized design, complete participant retention, and use of validated measurement tools.

### 4.1 Limitations

This study has several limitations. First, variability in acupressure technique and adherence may have occurred despite training and follow‐up, as self‐administration was involved. Second, the relatively small sample size and single‐center design may restrict generalizability. Third, the intervention and follow‐up period was limited to 4 weeks, leaving long‐term effects unknown. Finally, while efforts were made to blind participants and minimize placebo effects, the control condition involving light touching near acupoints might have induced minor therapeutic effects, potentially affecting comparisons.

## 5 Conclusion

n general, the results of our study indicate that acupressure can be effective in reducing anxiety and fatigue and improving sleep quality among operating room staff. Given the importance of mental and physical health for individuals working in the operating room, acupressure represents a feasible and non‐pharmacological complementary therapy. Clinically, its use could enhance staff well‐being, and potentially improve patient care through better staff performance. Therefore, the use of complementary and traditional therapies such as acupressure is recommended.

In general, the results of our study indicate that acupressure can be effective in reducing anxiety and fatigue and improving sleep quality among operating room staff. Therefore, given the importance of the mental and physical health of individuals working in the operating room, the use of complementary and traditional therapies such as acupressure is recommended.

## Author Contributions

**Rana Abjar:** conceptualization, investigation, writing – original draft, writing – review and editing, software, data curation, methodology, validation, project administration, resources, funding acquisition. **Ebrahim NasiriFormi:** conceptualization, investigation, funding acquisition, writing – original draft, methodology, validation, writing – review and editing, formal analysis, project administration, data curation, supervision. **Amir Hooman Kazemi:** investigation, methodology, validation, writing – original draft, formal analysis, writing – review and editing, supervision, data curation. **Hooshang Akbari:** investigation, writing – original draft, formal analysis, software, writing – review and editing, visualization. **Leila Sadati:** writing – original draft, writing – review and editing, software, formal analysis, investigation.

## Ethics Statement

This study received ethical approval from the Institutional Ethics Committee. IR.MAZUMS.REC.1398.1242.

## Conflicts of Interest

The authors declare no conflicts of interest.

## Transparency Statement

Dr. Ebrahim Nasiri Formi affirms that this manuscript is an honest, accurate, and transparent account of the study being reported; that no important aspects of the study have been omitted; and that any discrepancies from the study as planned (and, if relevant, registered) have been explained.

## Data Availability

The data that support the findings of this study are available from the corresponding author upon reasonable request. The data supporting the findings of this study can be obtained from the corresponding author upon reasonable request. Dr. Ebrahim Nasiri Formi and Rana Abjar had full access to all of the data in this study and takes complete responsibility for the integrity of the data and the accuracy of the data analysis.
